# Interleukin-33 promotes inflammation-induced lymphangiogenesis via ST2/TRAF6-mediated Akt/eNOS/NO signalling pathway

**DOI:** 10.1038/s41598-017-10894-x

**Published:** 2017-09-06

**Authors:** Longhui Han, Minglian Zhang, Xu Liang, Xin Jia, Jinchen Jia, Miying Zhao, Yiming Fan

**Affiliations:** 1 Hebei Provincial Key Laboratory of Ophthalmology, Hebei Provincial Eye institute, Hebei Provincial Eye Hospital, Xingtai, Hebei, 054001 China; 2 Tianjin Eye Hospital, Tianjin, 300020 China; 3 Tianjin Medical University Eye Hospital/Eye Institute, School of Optometry and Ophthalmology, Tianjin Medical University, Tianjin, 300384 China

## Abstract

The interplay between inflammation and lymphangiogenesis is mediated by various cytokines. However, most of these molecules and their associated mechanism are yet to be defined. Here, we explored the role of IL-33 in modulating inflammation-induced lymphangiogenesis (ILA) and its underlying mechanisms using an ILA mouse model and a lymphatic endothelial cell (LEC) line. Our results show that IL-33 promoted the proliferation, migration and tube formation of LECs and ILA *in vivo*. The pro-lymphangiogenic activity of IL-33 was abolished by ST2 blockage. In mechanisms, IL-33 induced the phosphorylation of Akt/eNOS to produce NO in LECs. The IL-33-induced Akt/eNOS activation was suppressed by the PI3K-specific-inhibitor wortmannin, and NO-production was inhibited by both wortmannin and the NO synthase-inhibitor NMA. Knock-down of ST2 or TRAF6 suppressed Akt/eNOS phosphorylation and NO production. The reduction of NO treated with wortmannin or NMA abolished the promoting effects of IL-33 on the chemotactic motility and tube formation of HDLECs. *In vivo*, IL-33-induced ILA was also impaired in eNOS^−/−^ mice. In conclusion, our study is the first to show that IL-33 promotes inflammation-induced lymphangiogenesis via a ST2/TRAF6-mediated Akt/eNOS/NO signalling pathway. This findings may provide us more opportunities to treat inflammation and lymphangiogenesis associated diseases.

## Introduction

Lymphangiogenesis has been implicated in many inflammatory processes^1^. Modulating lymphangiogenesis may provide more possibilities to treat inflammation-associated diseases^2^. Interplay between inflammation and lymphangiogenesis is mediated by various cytokines. However, most of these molecules and their associated mechanism are yet to be defined^3^.

IL-33 is a multifunctional cytokine and is the 11^th^ identified IL-1 family member^4^. IL-33 is localized in cell nucleus but also functions as a cell-free cytokine, and in this way is similar to HMGB1 and IL-1α^5–8^. IL-33 signals via the ST2 receptor, an orphan receptor that does not bind to IL-1α, IL-1β or other IL-1 family members^9^. IL-33 is mainly involved in type-2 immunity and inflammation and plays a key role in some pathological processes^7^. Although IL-33 was historically isolated from endothelial cells, epithelial cells, and keratinocytes, it is now recognized to be released from various tissues as a pro-inflammatory cytokine^4, 8, 10^.

Nitric oxide (NO) plays a crucial role in regulating the growth and function of lymphatic vessels^11, 12^. NO production in lymphatic endothelial cells (LECs) relies on the endothelial NO synthase (eNOS) activity, which is regulated by multiple inflammatory factors^11–15^. According to a recent study, eNOS inhibition blocks NO production and lymphangiogenesis^11^. Although the mechanisms by which NO regulates lymphangiogenesis are not completely understood, NO has emerged as an important mediator of physiological and pathological lymphangiogenesis and inflammation.

Since the identification of IL-33 as a functional ligand of ST2, the immunomodulatory and inflammatory roles of IL-33 have been well studied. However, the role of IL-33 in inflammation-associated lymphangiogenesis has not been determined. IL-33 is a pro-inflammatory cytokine and also induces eNOS/NO activation. Inflammation and eNOS/NO activation are closely related to lymphangiogenesis. Thus, we hypothesized that IL-33 has a regulatory function in inflammation-associated lymphangiogenesis via the eNOS/NO signalling pathway. To test this hypothesis, we used a mouse model of ILA and a LEC cell line. The results showed that IL-33 promoted the proliferation, migration and tube formation of LECs *in vitro* and inflammation-induced lymphangiogenesis (ILA) *in vivo*, which were regulated by PI3K/Akt/eNOS-mediated NO production. ST2/TRAF6 was required for IL-33-induced Akt/eNOS activation and NO production.

Our results indicate for the first time that IL-33 promotes inflammation-induced lymphangiogenesis via the ST2/TRAF6-mediated Akt/eNOS/NO signalling pathway. This findings may provide us more opportunities to treat inflammation and lymphangiogenesis associated diseases.

## Results

### IL-33 promotes the proliferation, migration and tube formation of LECs via the ST2 receptor

**Figure 1:**
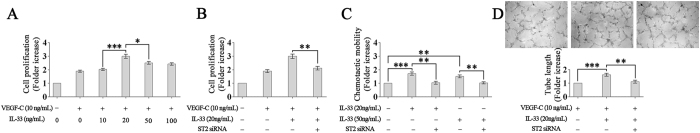
IL-33 promotes LECs proliferation, migration and tube formation via the ST2 receptor. (**A**) IL-33 promotes IL-33-induced HDLECs proliferation in a dose-dependent manner. (**B**) The ST2 receptor mediates IL-33-induced HDLECs proliferation. (**C**) The ST2 receptor mediates IL-33-induced HDLECs chemotactic mobility. (**D**) The ST2 receptor mediates IL-33-induced HDLECs tube formation. Three independent experiments were performed in duplicate. *p < 0.05, **p < 0.01, ***p < 0.001.

To explore the lymphangiogenic activity of IL-33, we first determined whether IL-33 promoted LECs proliferation *in vitro*. Our results showed that IL-33 promoted VEGF-C–induced HDLECs proliferation in a dose-dependent manner, with a maximal effect at 20 ng/mL (Fig. 1A). Knock-down of ST2 with an ST2-specific siRNA significantly abolished the promoting effect of IL-33 (Fig. 1B), indicating that ST2 mediates IL-33-induced LECs proliferation.

Then, we tested the roles of IL-33 and ST2 in LECs chemotactic mobility and tube formation. The results showed that IL-33 promoted chemotactic mobility and increased the tube length, and these effects were inhibited by ST2 knock-down (Fig. 1C and D).

IL-33 itself (without additional VEGF-C stimulation) also induced LECs proliferation and tube formation (Figure S2). In addition, IL-33 didn’t up-regulate the expression of pro-lymphangiogenic factors (VEGF-C/D) by LECs (Figure S2).

So, we can confirm that it is IL-33 itself, not indirect VEGF-C/D, that takes the main effects on LECs in the test.

### IL-33 promotes ILA in the mouse cornea via the ST2 receptor

**Figure 2:**
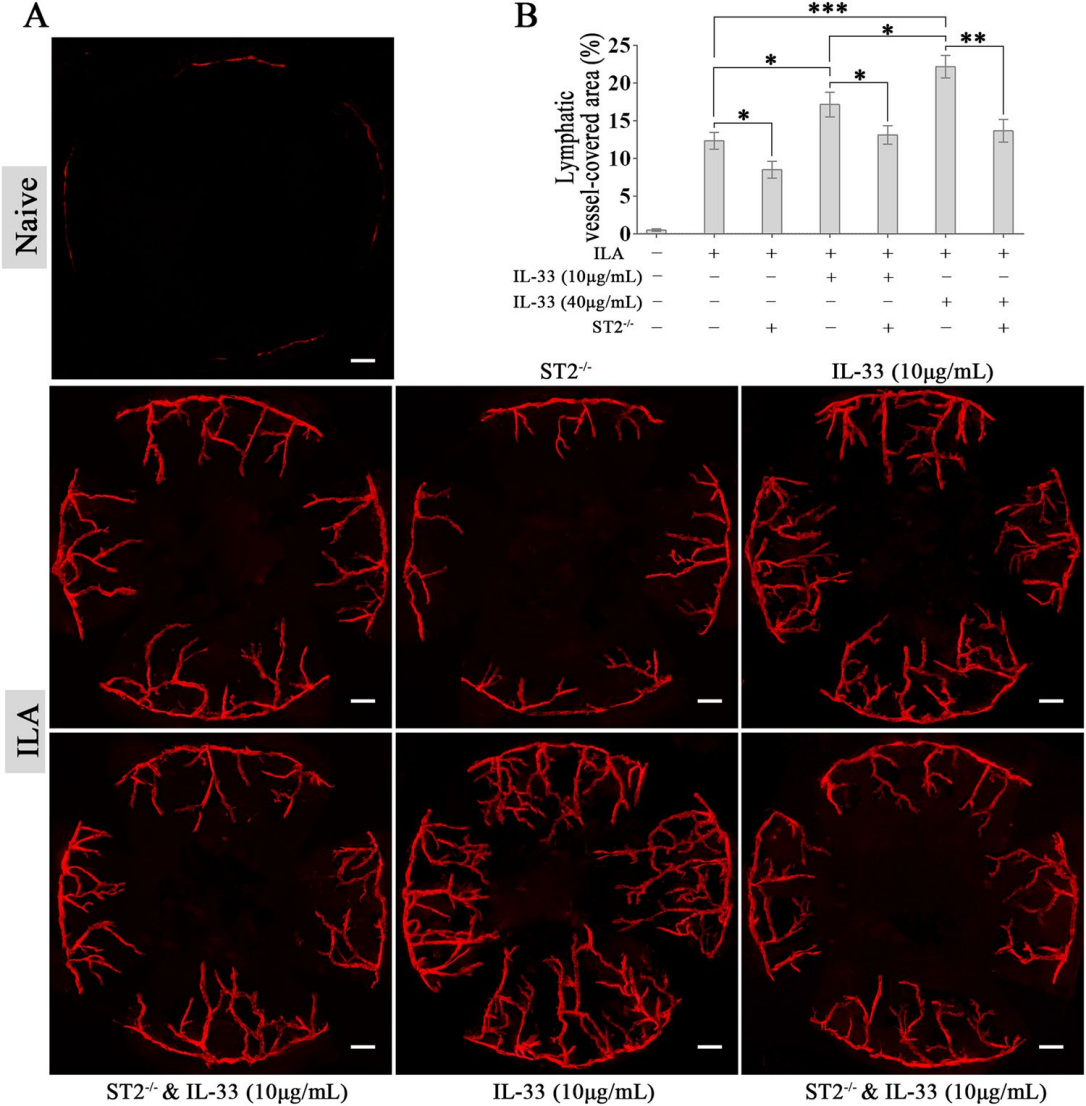
IL-33 promotes ILA in the mouse cornea via the ST2 receptor. (**A**,**B**) Representative images and quantification of LYVE-1-labelled corneal lymphangiogenesis in different groups showing that the ST2 receptor mediates IL-33-associated ILA. Three independent experiments were performed in duplicate. *p < 0.05, **p < 0.01. The scale bars represent 300 μm.

*In vivo*, a lower concentration (10 μg/mL) of IL-33 treatment promoted the ILA in mouse corneas (Fig. 2A and B). A higher concentration (40 μg/mL) of IL-33 induced more ILA. ST2 knock-out decreased ILA in both the presence (low or high concentrations) and absence of IL-33. These results indicate that IL-33/ST2 signalling does take effects in promoting ILA.

### IL-33 stimulates NO production in LECs via the PI3K/Akt/eNOS signalling pathway

**Figure 3:**
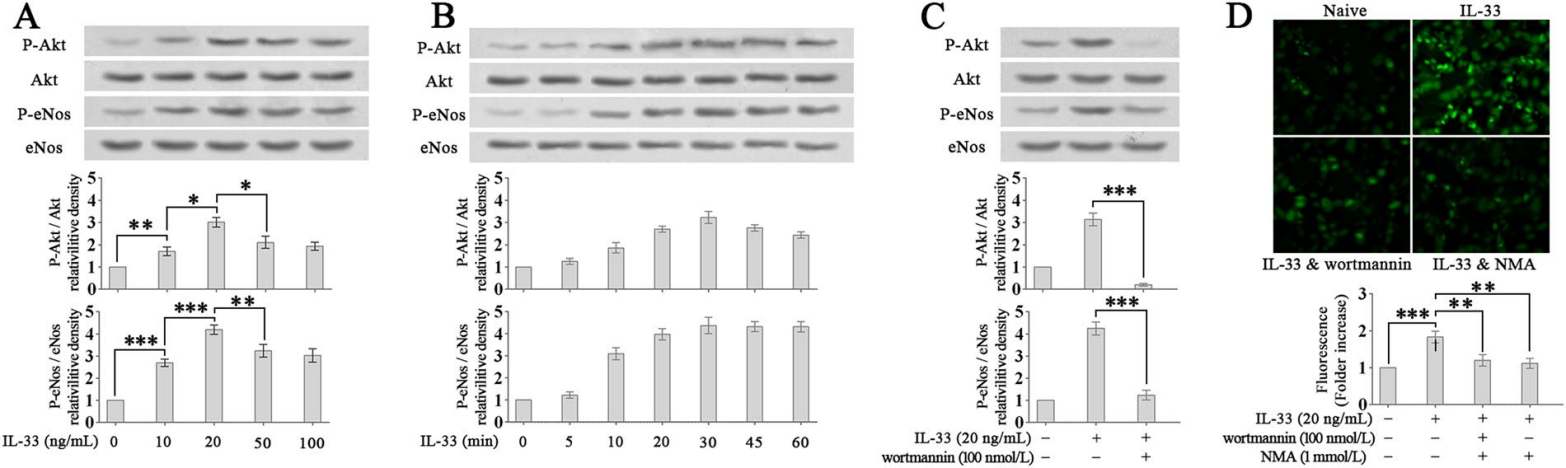
IL-33 stimulates NO production in LECs via the PI3K/Akt/eNOS signalling pathway. (**A**–**C**) IL-33-induced (20 ng/mL) Akt and eNOS phosphorylation was determined by Western blotting. (**A**) HDLECs were stimulated with various concentrations of IL-33 for 30 min. (**B**) HDLECs were stimulated with IL-33 (20 ng/mL) for the indicated times. (**C**) HDLECs were pre-treated with wortmannin (100 nmol/L, 30 min) and then stimulated with IL-33 (20 ng/mL, 30 min). (**D**) HDLECs were pre-treated with wortmannin (100 nmol/L, 30 min) or NMA (1 mmol/L) and then treated with IL-33 (20 ng/mL) for 4 hours. LECs were incubated with DAF-FM DA for 1 hour at 37 °C. The relative intracellular NO levels were determined from the fluorescence intensity of DAF-FM. Three independent experiments were performed in duplicate. *p < 0.05, **p < 0.01, ***p < 0.001. Full-length blots are presented in Supplementary Figure S3.

NO plays a crucial role in regulating the growth and function of lymphatic vessels^11, 12^. NO production in the lymphatic system is usually regulated by PI3K/Akt/eNOS signaling^16–19^. Therefore, we tested whether IL-33 induces PI3K/Akt/eNOS activation to produce NO in LECs. Western blotting showed that IL-33 promoted the phosphorylation of Akt and eNOS, with a maximal effect at 20 ng/mL (Fig. 3A). IL-33-induced Akt and eNOS phosphorylation began to increase significantly at 10 min after treatment and was sustained for at least 50 min (Fig. 3B).

Further, we investigated whether PI3K was required for the activation of Akt/eNOS using wortmannin (a PI3K-specific inhibitor). As a result, the wortmannin treatment (100 nmol/L, 30 min) limited IL-33-induced Akt and eNOS phosphorylation to a very low levels, indicating that PI3K is required for IL-33-induced Akt/eNOS activation (Fig. 3C).

IL-33-induced NO production was also suppressed by the wortmannin or NMA (a NO synthase inhibitor) treatment (Fig. 3D).

### ST2/TRAF6 is required for IL-33-induced Akt/eNOS activation and NO production

**Figure 4:**
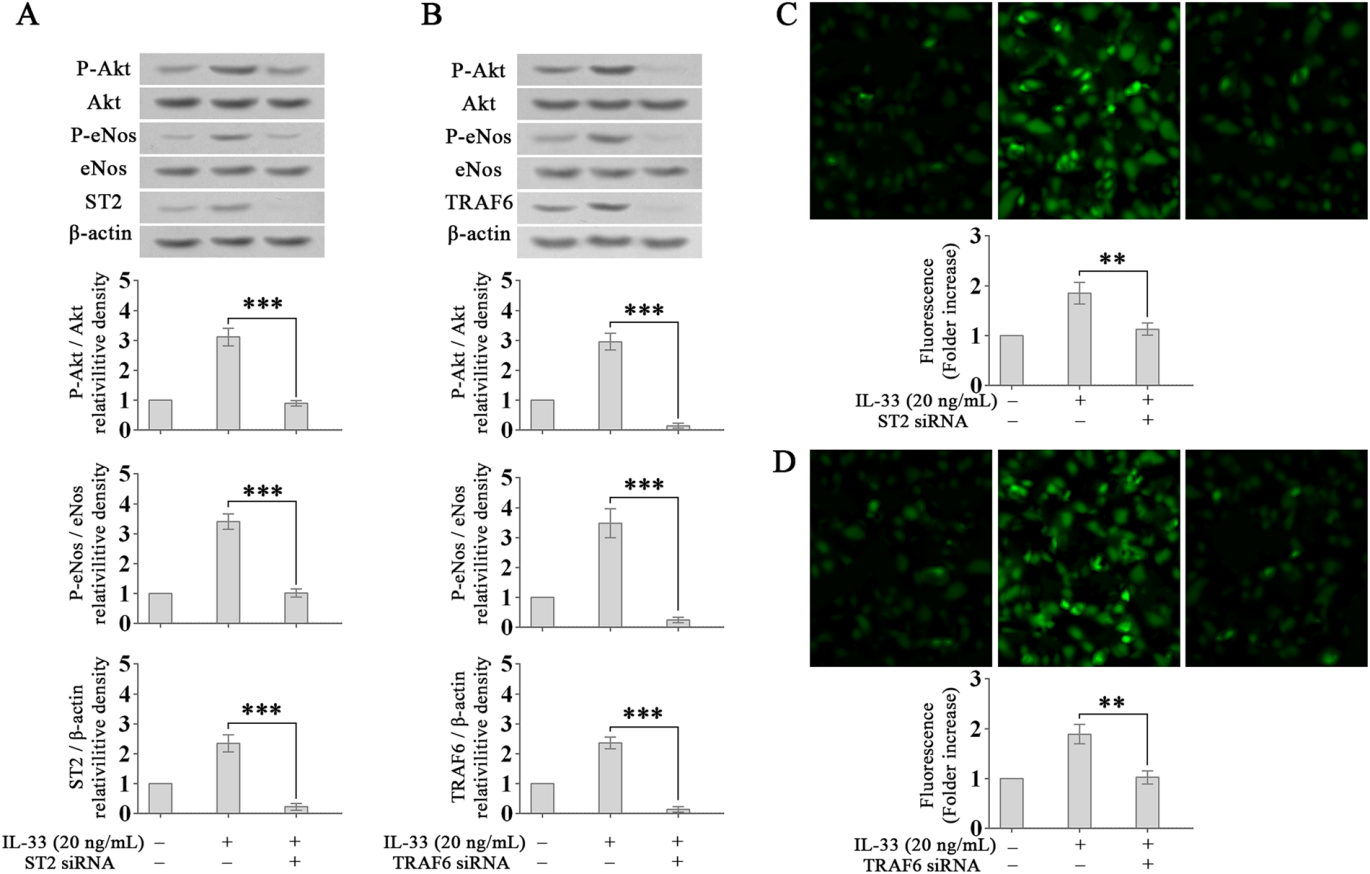
IL-33 induces Akt/eNOS activation and NO production via ST2/TRAF6. (**A**,**B**) After transfection with an ST2- or TRAF6-specific siRNA, HDLECs were treated with IL-33 (20 ng/mL, 30 minutes). Akt and eNOS phosphorylation were detected by Western blotting. (**C**,**D**) After transfection with the ST2- or TRAF6-specific siRNA, HDLECs were treated with IL-33 (20 ng/mL, 4 hours) and incubated with DAF-FM DA for 1 hour. Relative intracellular NO levels were determined from the fluorescence intensity of DAF-FM. Three independent experiments were performed in duplicate. **p < 0.01, ***p < 0.001. Full-length blots are presented in Supplementary Figure S4.

TRAF6 has been reported to mediate Akt/eNOS activation and is modulated by ST2^20–22^. Our results showed that the elevated ST2 or TRAF6 expression induced by IL-33 increased Akt/eNOS phosphorylation (Fig. 4A and B). On the other hand, the knock-down of ST2 or TRAF6 by an ST2- or TRAF6-specific siRNA suppressed Akt/eNOS phosphorylation and NO production (Fig. 4A–D). Thus, the results suggest that ST2 and TRAF6 are upstream regulators of IL-33-induced Akt/eNOS activation.

Taken together, the above results demonstrate that IL-33 promotes the NO production in LECs via a ST2/TRAF6-PI3K/Akt/eNOS signalling pathway.

### PI3K/Akt/eNOS-mediated NO production is required for IL-33-induced ILA

**Figure 5:**
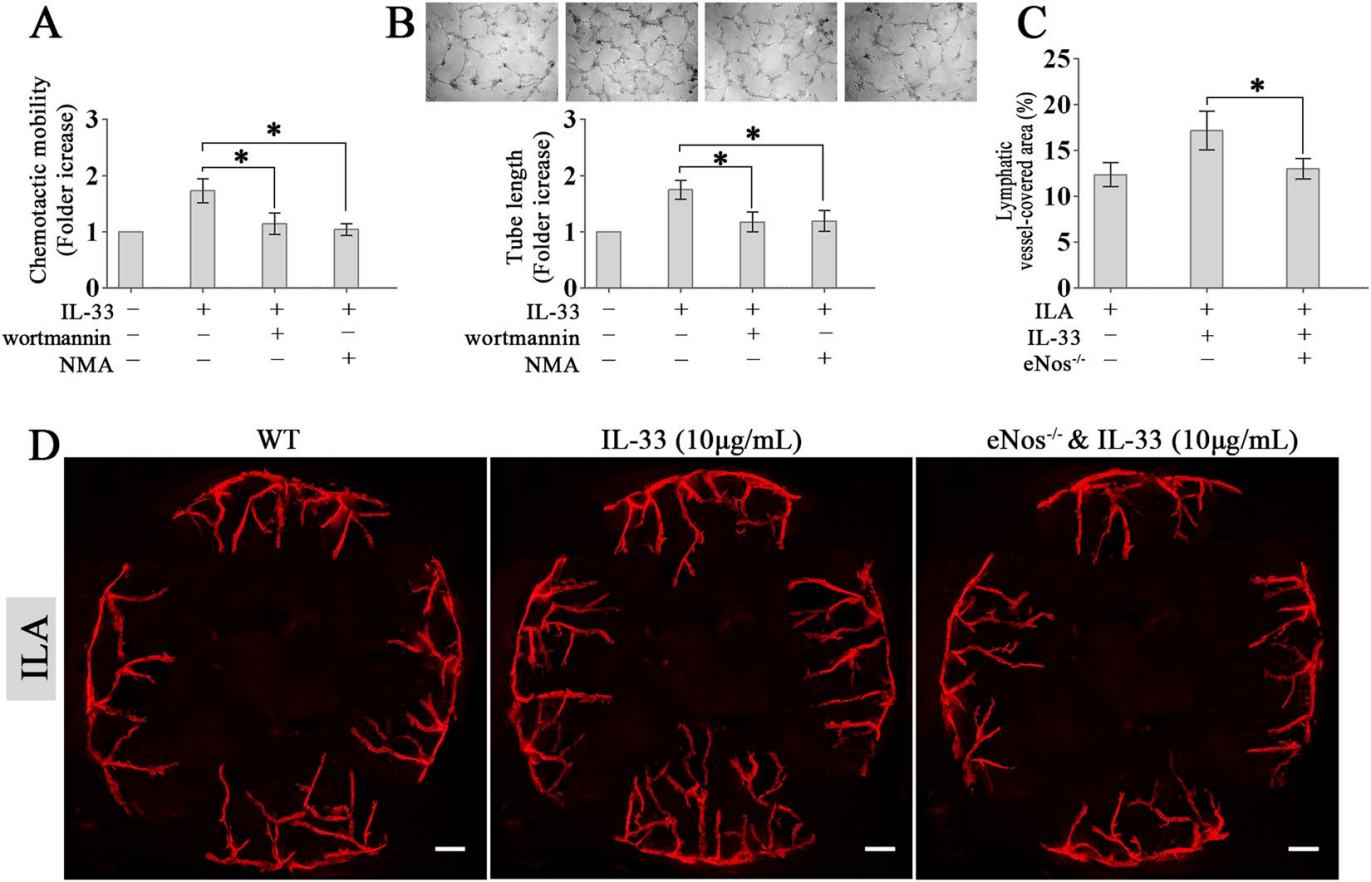
PI3K/Akt/eNOS-mediated NO production is required for IL-33-induced ILA. (**A**,**B**) HDLECs were pre-treated with or without wortmannin (100 nmol/L) or NMA (1 mmol/L) for 30 minutes before stimulation with IL-33 (20 ng/mL). (**A**) Chemotaxis was quantified after a 4 hour incubation. (**B**) HDLECs tube formation was quantified after a 16 hour incubation. (**C**) After IL-33 (10 μg/mL) treatment, ILA was quantified in WT and eNOS KO mice *in vivo*. Three independent experiments were performed in duplicate. *p < 0.05.

To evaluate the role of PI3K/Akt/eNOS-mediated NO production in IL-33-induced ILA, HDLECs were treated with wortmannin or NMA before IL-33 stimulation and then the chemotactic motility and tube formation of HDLECs were assessed. The reduction of NO production following treatment with wortmannin or NMA abolished the promoting effects of IL-33 on HDLECs chemotactic motility and tube formation (Fig. 5A and B). *In vivo*, IL-33-induced ILA was also impaired in eNOS^−/−^ mice compared with WT mice (Fig. 5C). These results show that PI3K/Akt/eNOS-mediated NO production is required for IL-33-induced ILA.

## Discussion

In the present study, we explored the role of IL-33 in inflammation-induced lymphangiogenesis and its associated mechanisms. For the first time, we show that IL-33 directly activates LECs, resulting in promoting inflammation-induced lymphangiogenesis. Inflammation and lymphangiogenesis are associated with multiple diseases; therefore, our findings may provide us more opportunities to treat inflammation and lymphangiogenesis associated diseases.

Firstly, we find that IL-33 is involved in ILA (Figure S1). Both mRNA and protein of IL-33 are significantly increased in the inflamed corneas after the ILA surgery. This finding is consistent with the results reported by Hazlett LD, who showed IL-33 mRNA levels were significantly up-regulated in both BALB/c and B6 mouse corneas after infection, and immuno-staining used to localize IL-33 in the cornea showed qualitatively intense IL-33-positive staining^23^. Therefore, a topical blockade of IL-33 would be a possible treatment for corneal lymphangiogenesis-associated diseases.

To determine the lymphangiogenic activity of IL-33, we tested whether IL-33 directly activated LECs. Our *in vitro* tests verified that IL-33 does not up-regulate the expression of pro-lymphangiogenic factors (VEGF-C/D) in LECs, and IL-33 itself promotes the proliferation, migration and tube formation of LECs through a VEGF-C/D-independent mechanism (Figs 1 and S2). Most importantly, our further *in vivo* tests ascertain that IL-33 promotes inflammation-induced lymphangiogenesis in the mouse cornea. IL-33 takes effect mainly via the ST2 receptors^5, 24, 25^. When we blocked ST2, most of the pro-lymphangiogenic activity of IL-33 was abolished both *in vitro* and *in vivo*, which confirms the pro-lymphangiogenic role of IL-33.

Our repeated tests show that the effects of IL-33 on LECs were reduced when cells were treated with concentrations greater than 20 ng/mL. A similar phenomenon was also observed in some other studies^21, 26^. According to Choi YS, *et al*., IL-33 stimulated the proliferation, chemotactic motility and tube formation of HUVECs, with a maximal effect at 20 ng/mL^21^, and these effects decreased at 50 ng/mL. As shown in the study by Hayashi H, *et al*., IL-33 enhanced fibrocyte proliferation, with a maximal effect at 10 ng/mL, and the number of viable fibrocytes decreased at 100 ng/mL^26^. Higher concentrations of IL-33 than the concentrations that exert the maximal effects on LECs may induce inhibitory mechanisms. For example, Hazlett LD, *et al*. showed that overexpression of IL-33 markedly decreased the expression of pro-inflammatory cytokines (IL-1, MIP-2, IL-6, and TNF-alpha)^23^. Other as yet unknown mechanisms may also contribute. We may explore these mechanisms in the future.

We did not notice a substantial difference in ILA between untreated WT group and IL-33 treated ST2^−/−^ groups. This result suggests that ST2 knock-out only blocks partial, but not all, effects of IL-33 on ILA. The study by Luzina IG reported a similar phenomenon and their results showed that ST2 knock-out attenuated only the size of, but did not completely abrogate, mature-mouse-IL-33-induced inflammatory infiltrates^25^. Their further tests certificates that some of the effects of IL-33 on the lung were mediated by ST2-independent mechanisms, such as up-regulating the expression of CCL2 (MCP-1), IL-6, and matrix metalloproteinases (MMPs) 3, 10, and 13 genes. These ST2-independent mechanisms may have been responsible for the lack of a difference in ILA between untreated WT group and IL-33 treated ST2^−/−^ groups. We may verify this hypothesis in the future.

During these experiments, we observed a interesting phenomenon that exogenous IL-33 up-regulated ST2 expression in LECs. Furthermore, some other researches showed the similar phenomenon. As shown in the study by Hazlett LD, increased ST2 staining is associated with microphages in IL-33–treated mice, and ST2 mRNA is significantly up-regulated in microphages *in vitro* after LPS stimulation in the presence of exogenous IL-33^23^. According to Choi YS, exogenous IL-33 up-regulates ST2 expression in HUVECs^21^. We still do not know the exact mechanisms underlying this phenomenon. However, we have proposed some possible hypotheses. For example, some positive feedback mechanisms that promote inflammation will occur in the early-stage of inflammation. The gradually increased inflammation-associated factors stimulates LECs to increase ST2 expression. The phenomenon could be completely illustrated in the future.

Several previous studies have reported a role for IL-33 in mediating angiogenesis^21, 27–31^. Most of these studies support the hypothesis that IL-33 promotes angiogenesis directly through its pro-angiogenic effects^21^ or indirectly by up-regulating pro-angiogenic factors^27–30^, such as VEGF, angiogenin, von Willebrand factor (vWF), etc. Only one paper draws the opposite conclusion that IL-33 inhibits angiogenesis, but the investigators did not show the exact mechanisms. Although researchers hold different opinions, all agree that ST2 is dependent for IL-33-mediated angiogenesis. In our study, IL-33 promotes lymphangiogenesis in a VEGF-C/D-independent manner, consistent with the results reported by Choi YS^21^.

In addition, accumulating evidence indicates that IL-33/ST2 has emerged as an intercellular signalling pathway that participates in various processes, such as autoimmunity, antigen/allergen responses, organ fibrosis, cardiac injury and a variety of other inflammatory conditions^5, 24^. Efforts to clarify the role of IL-33/ST2 in the interaction between end-organ effector cells and their environment may result in the discovery of novel therapeutic targets for the modulation of the above pathological conditions.

NO plays crucial role not only in regulating blood vessel growth and function but also in regulating lymphangiogenesis^11, 21^. NO donors stimulate lymphatic endothelial cell proliferation, migration and tube formation^11, 12, 14, 15^. NOS inhibition blocks lymphangiogenesis in a dermal regeneration mouse-tail model^11^. NO production during lymphangiogenesis is usually regulated by PI3K/Akt/eNOS signaling^16–19^. Therefore, we performed additional experiments to explore the PI3K/Akt/eNOS signalling associated mechanisms in IL-33-induced lymphangiogenesis. Our results showed that IL-33 induced Akt/eNOS activation to produce NO in LECs. IL-33-induced Akt and eNOS phosphorylation was suppressed by the PI3K-specific inhibitor wortmannin. Furthermore, NO production was inhibited by both wortmannin and the NO synthase inhibitor NMA. These findings suggests that IL-33 stimulates NO production in LECs via the PI3K/Akt/eNOS signalling pathway.

TNF-receptor associated factor 6 (TRAF6) is a critical signal transducer in the IL-33 signalling pathway^21, 22^. TRAF6 inhibition suppressed Akt/eNOS activation and NO production in endothelial cells (ECs)^20, 21^. TRAF6 is also expressed in LECs^32^. Therefore, the possibility is raised that IL-33 may induce LECs activation via TRAF6-mediated eNOS/NO pathway. Our present study shows that the elevated TRAF6 expression induced by IL-33 increased the Akt/eNOS phosphorylation, and the knock-down of TRAF6 with a TRAF6-specific siRNA suppressed Akt/eNOS phosphorylation and NO production, which confirms that TRAF6 is a crucial bridge that mediates IL-33/ST2 and PI3K/Akt/eNOS/NO signalling in LECs.

Finally, we performed additional experiments to evaluate the role of PI3K/Akt/eNOS-mediated NO production in IL-33-induced ILA. Our *in vitro* tests showed that the reduction of NO by using PI3K-specific inhibitor wortmannin or NOS inhibitor NMA abolished the effect of IL-33 on HDLECs chemotactic motility and tube formation. Our *in vivo* tests showed that the knock-out of eNOS suppressed ILA induced by IL-33. These results confirms that PI3K/Akt/eNOS-mediated NO production is required for IL-33-induced ILA.

In conclusion, to our knowledge, the present study provides the first evidence that IL-33 promotes inflammation-induced lymphangiogenesis via ST2/TRAF6-mediated Akt/eNOS/NO signaling pathway. Consequently, the administration of modulating inflammation-induced lymphangiogenesis through IL-33 and its downstream signalling TRAF6-Akt/eNOS/NO may provide us more possibilities of treating inflammation and lymphangiogenesis associated diseases.

## Materials and Methods

### Ethics statement and animals

The study strictly adhered to the Association for Research in Vision and Ophthalmology’s Statement for the Use of Animals in Ophthalmic and Vision Research. All animal experiments were conducted with the approval and supervision of the Institutional Animal Care and Use Committee of Hebei Provincial Eye Hospital. Eight-week-old male C57BL/6 mice, ST2^−/−^ mice on the C57BL/6 background and eNOS^−/−^ mice on the C57BL/6 background were obtained from the Model Animal Research Center of Nanjing University. All surgeries were performed under chloral hydrate anaesthesia.

### Antibodies and reagents

The antibodies, including polyclonal anti-lymphatic vessel endothelial hyaluronan receptor-1 (LYVE-1), monoclonal anti-Akt, monoclonal anti-phosphorylated Akt, monoclonal anti-eNOS, and monoclonal anti-phosphorylated eNOS antibodies, were obtained from Abcam (Hong Kong, China). Other antibodies, including the monoclonal anti-β-actin antibody, horseradish peroxidase (HRP)-conjugated anti-mouse secondary antibody, HRP-conjugated anti-rabbit secondary antibody, and Alexa Fluor 555-conjugtaed goat anti-rabbit secondary antibody, were purchased from Cell Signaling Technology Inc. (Danvers, MA). Recombinant mouse IL-33 was purchased from Alexis Biochemicals (San Diego, USA). VEGF-C was obtained from Sigma (St. Louis, MO, USA). Wortmannin and N^G^-Monomethyl-L-arginine (NMA) were obtained from Abcam (Hong Kong, China).

### Cell culture

Human dermal lymphatic endothelial cells (HDLECs) were purchased from ScienCell (Carlsbad, CA, USA) and grown in endothelial cell basal medium-2 with growth supplements (EBM-2 MV) at 37 °C in a humidified 95%/5% (vol/vol) air/CO_2_ atmosphere.

### LEC proliferation assay

For the proliferation assay, HDLECs were incubated with different doses of IL-33 (0, 10, 20, 50 or 100 ng/mL) with or without VEGF-C (10 ng/mL) for 24 hours. Then, 100 μL of cells from each well were transferred to a new 96-well plate and incubated with 10 μL of Cell Counting Kit-8 solution (Dojindo Laboratories, Kumamoto, Japan). The absorbance was measured at 450 nm using a microplate reader.

### LEC migration assay

Transwell chambers (Corning Costar) were used to assay the chemotatic motility of HDLECs. The 6.5 mm diameter polycarbonate filters (8 μm pore size) were used. The lower surface of the filter was coated with 10 μg of gelatine. Fresh EBM-2 MV medium containing IL-33 (20 or 50 ng/mL) was placed in the lower wells. The cells were suspended at a final density of 10^6^ cells/mL in EBM-2 MV medium. Cell suspensions (100 μL) were loaded into each of the upper wells and incubated at 37 °C for 4 hours. The cells were fixed and stained with haematoxylin and eosin. Cells on the upper surface of the filter were removed, and chemotaxis was quantified by counting the cells that had migrated to the lower side of the filter under an optical microscope.

### Tube formation assay

Tube formation was assayed using a previously described method^33, 34^. Mixtures of 30 μL of Matrigel (BD Biosciences) and 20 μL of EBM-2 were polymerized in each well of a 96-well plate. Approximately 6 × 10^3^ HDLECs in 100 μL of ECM were placed on the Matrigel layer in each well. After a 16-hour incubation at 37 °C, tube morphogenesis was assessed using phase contrast microscopy. The total tube length was quantified using ImageJ software (National Institutes of Health).

### Knock-down of ST2 or TRAF6 by siRNA transfection

HDLECs cultured in a 6-well plate were transfected with 180 pmol of ST2- or TRAF6-specific siRNAs (Santa Cruz Biotechnology, Inc., Santa Cruz, USA). After 48 hours of transfection, cells were used for further experiments.

### ILA model and treatment

An ILA model was established using previously described methods^35–37^. Briefly, a 2-mm corneal trephine was used to mark a circle on the centre of the cornea in the right eye. Three “8”-shaped intrastromal sutures were performed using 11-0 nylon sutures (Jiahe Inc., Taibei, Taiwan, China; NT0411), and each suture extended to approximately 120° of the corneal circumference. Then, the ILA model mice were randomly divided into each group (n = 5) and were administered a topical treatment of 5 μL IL-33 (10 or 40 μg/mL) or vehicle (sterile saline solution) in the right eye twice a day for 10 days. The *in vivo* experiments were independently repeated 3 times.

### Immunostaining and quantification

Whole-mount corneal LYVE-1 immunostaining was performed as previously described^35, 38^. Briefly, the mice were sacrificed on the 10th day. The eyeballs were fixed with 4% (wt/vol) paraformaldehyde for 16 hours at 4 °C. The excised corneas were blocked with 3% bovine serum albumin (BSA) in PBST (0.3% (vol/vol) Triton X-100 in PBS) for 2 hours, followed by an overnight incubation at 4 °C with the anti-LYVE-1 antibody (1:200). On the second day, the tissue was incubated with an Alexa Fluor 555-conjugated goat anti-rabbit antibody (1:400) for 2 hours at room temperature. The cornea was flat-mounted on microscope slides with Vectashield mounting medium and then examined under a fluorescent microscope. Lymphatic vessels were quantified using Adobe Photoshop CC software, as previously described^35^.

### Western blot analysis

HDLEC or cornea lysates (50 μg of total protein) were separated on a polyacrylamide-SDS gel and electro-blotted onto a nitrocellulose membrane (Bio-Rad, Hercules, CA, USA). After blocking with 5% non-fat milk, the membrane was incubated with antibodies against Akt, P-Akt, eNOS, P-eNOS, ST2, TRAF6 and β-actin (1:1000), followed by an incubation with an HRP-conjugated secondary antibody (1:3000). Signals were visualised using enhanced chemiluminescence detection (Pierce, Rockford, IL).

### Intracellular NO assay

Intracellular NO levels were assayed using a previously described method^21^. Briefly, HDLECs were treated with 20 ng/mL IL-33 for 4 hours and then incubated with 5 μmol/L 3-amino,4-aminomethyl-2′,7′-difluorescein, diacetate (DAF-FM DA) (Beyotime Biotechnology, China) for 1 hour at 37 °C. After the excess probe was removed, cells were incubated for an additional 20 minutes. Fluorescence images were captured from at least 5 randomly selected fields per dish using a fluorescence microscope. The intracellular NO level was determined from the fluorescence of DAF-FM.

### Statistical analysis

Data are presented as means ± SD. Statistical analyses were performed using SPSS 21.0 software (SPSS Inc., Chicago, IL). The normal distribution of the data was tested with Kolmogorov-Smirnov tests. Statistical comparisons between two groups were performed using Student’s t-tests. A *p* < 0.05 was considered statistically significant.

### Data Availability

The datasets generated and/or analysed during the current study are available from the corresponding author upon reasonable request.

## Electronic supplementary material

Supplementary information
