# Supplementary material for: Interleukin-33 promotes inflammation-induced lymphangiogenesis via ST2/TRAF6-mediated Akt/eNOS/NO signalling pathway
Source: Sci Rep. 2017 Sep 6;7:10602. doi: 10.1038/s41598-017-10894-x (PMC5587532; doi:10.1038/s41598-017-10894-x)
Supplement: Supplementary file 1 — Supplementary information [file 41598_2017_10894_MOESM1_ESM.pdf]

Interleukin-33 promotes inflammation-induced lymphangiogenesis via ST2/TRAF6-mediated Akt/eNOS/NO signalling pathway

Longhui Han[1,3#*], Minglian Zhang[1#*], Xu Liang[2#], Xin Jia[1#], Jinchen Jia[1#], Miying Zhao[1] & Yiming Fan[1]

[1] Hebei Provincial Key Laboratory of Ophthalmology, Hebei Provincial Eye institute, Hebei Provincial Eye Hospital, Xingtai, Hebei, 054001, China.

[2] Tianjin Eye Hospital, Tianjin, 300020, China.

[3] Tianjin Medical University Eye Hospital/Eye Institute, School of Optometry and Ophthalmology, Tianjin Medical University, Tianjin, 300384, China.

* Correspondence and requests for materials should be addressed to L.H. (email: han-longhui@163.com) or Minglian.Z. (zhmlyk@sohu.com)

SUPPLEMENTARY INFORMATION includes:

Figure S1, Figure S2 and full-length blots (Figure S3 and S4).

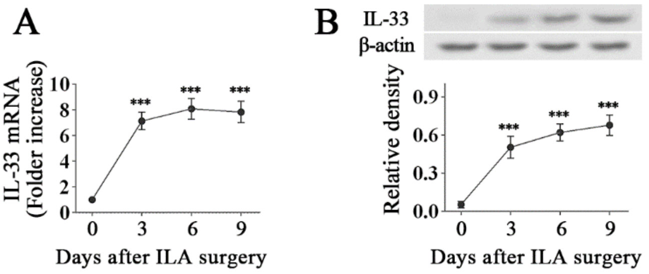

Figure S1. IL-33 is involved in ILA

(A): Real-time PCR shows the increased expression of IL-33 mRNA in the inflamed corneas after the ILA surgery. (B): Western blot shows the increased expression of IL-33 protein in the inflamed corneas after the ILA surgery. ***p < 0.001.

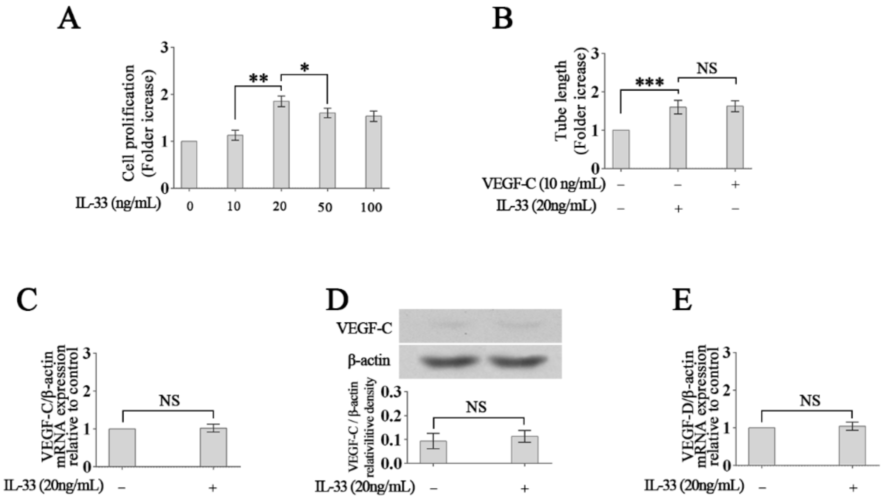

Figure S2. IL-33 increases the LECs proliferation and tube formation in a VEGF-C/D independent way

(A and B): IL-33 itself (without additional VEGF-C stimulation) also induces LECs proliferation and tube formation. (C-E): Real-time PCR and Western blot shows that IL-33 doesn’t up-regulate the expression of pro-lymphangiogenic factors (VEGF-C/D) by LECs. **p* < 0.05, ***p* < 0.01, ****p* < 0.001.

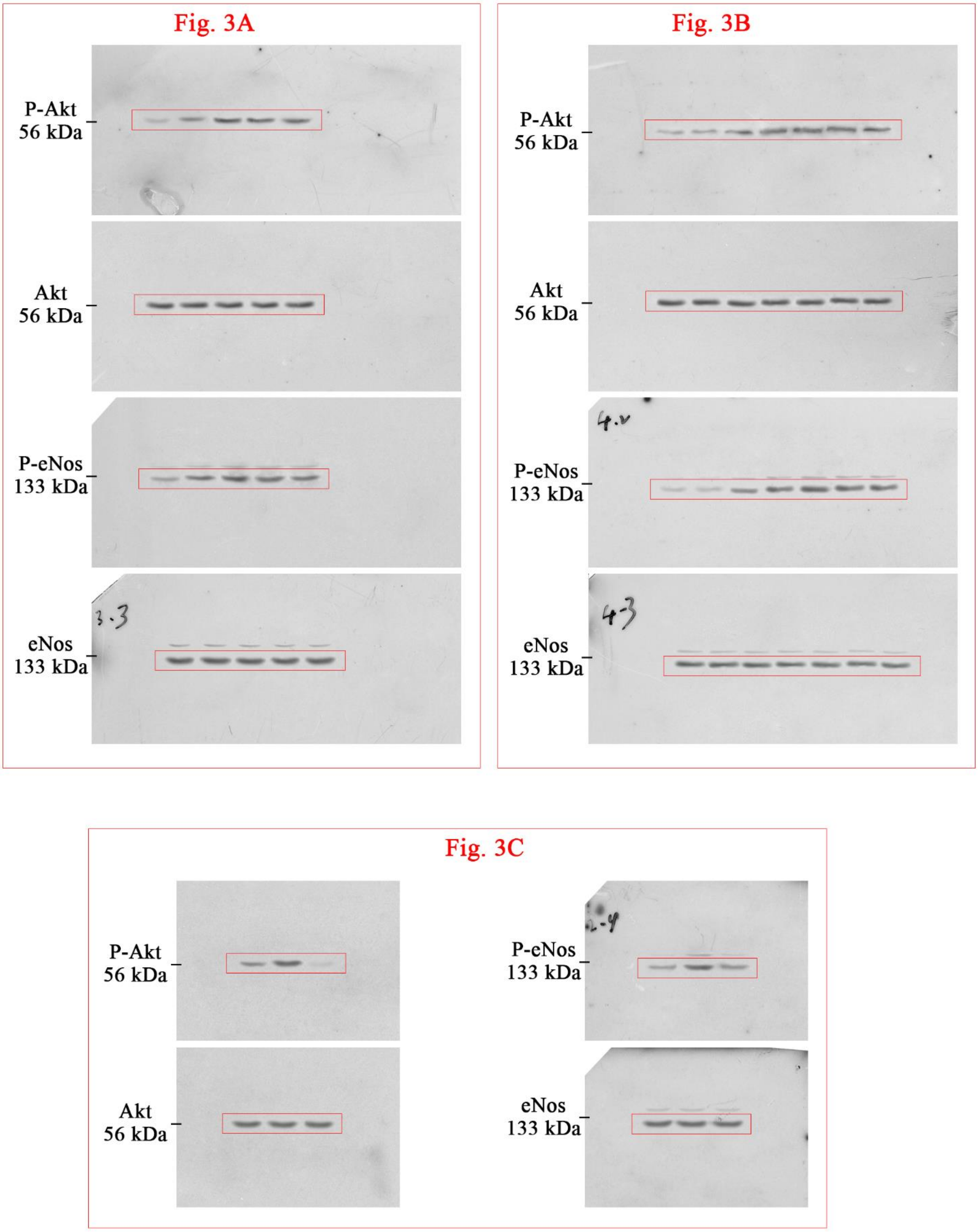

Figure S3 Full-length blots for fig. 3

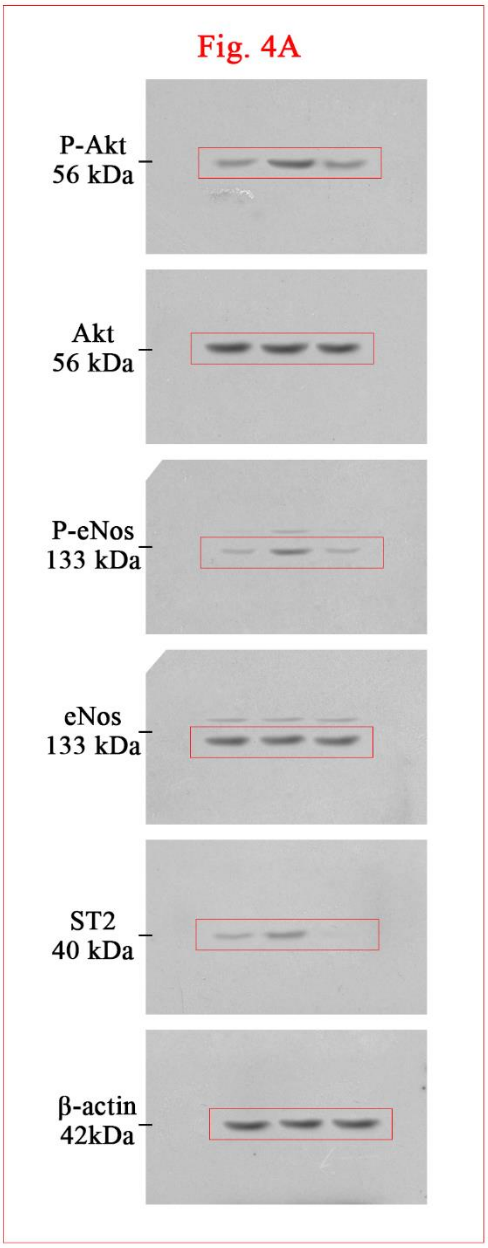

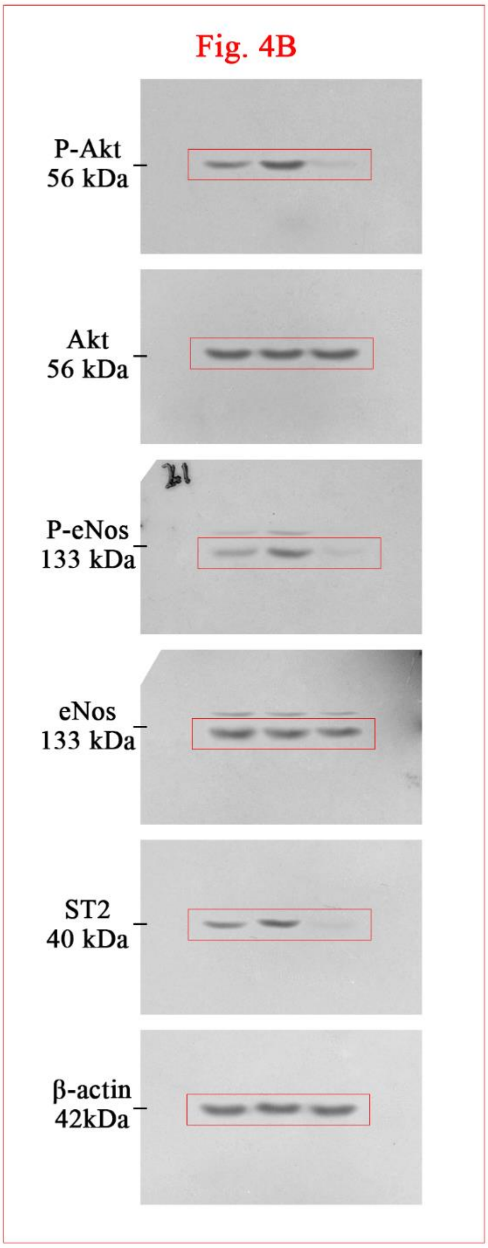

Figure S4 Full-length blots for fig. 4
